# Leiomyoma of scrotum: A rare case report

**DOI:** 10.1016/j.ijscr.2024.110490

**Published:** 2024-10-19

**Authors:** Mohadeseh Karimi, Hanieh Rad

**Affiliations:** Department of Pathology, Faculty of Medicine, Hormozgan University of Medical Sciences, Bandar Abbas, Iran

**Keywords:** Scrotum, Leiomyoma, Leiomyosarcoma

## Abstract

**Introduction and importance:**

Scrotal Leiomyoma is a benign and rare mesenchymal tumor that grows slowly. It was reported for the first time in 1858 by Forster. The Patients with Scrotal Leiomyoma is an asymptomatic painless mass.

**Case presentation:**

The patient was a 45-year-old man, who presented with a painless scrotal mass to our hospital. On physical examination, he had a single, firm, and mobile mass about 2–3 cm in diameter within the right scrotum without tenderness. The left side was normal. Pre-operative scrotal sonography, a 2.5 cm hypoechoic extra testicular mass with sharp borders was reported. Intraoperatively The mass was excised totally and the testis was preserved. On microscopic examination revealed interlacing bundles of spindle-shaped cells with no mitotic figures or nuclear pleomorphism. On immunohistochemical analysis, the spindle cells were positive for SMA and negative for S100, and leiomyoma was confirmed.

**Clinical discussion:**

For diagnosing leiomyoma, scrotal ultrasound is a first-line and noninvasive method that is confirmed by histologic examination. In histology, smooth muscle bundles are seen. Immunohistochemistry studies can confirm the diagnosis by identifying specific markers like vimentin, desmin, and smooth muscle actin. It is important to differentiate leiomyoma from a malignant type known as leiomyosarcoma.

**Conclusion:**

We report a rare case presentation of a 45-year-old man with scrotal leiomyoma that is confirmed by histology. It is important to differentiate leiomyoma from malignant course.

## Highlights

• Scrotal Leiomyoma is a benign and rare mesenchymal tumor.
• In histology, smooth muscle bundles are seen. It is important to differentiate leiomyoma from a malignant type known as leiomyosarcoma.

## 1 Introduction

Scrotal Leiomyoma is a benign and rare mesenchymal tumor. The most common site of leiomyoma is the uterus in reproductive-age women but the other locations, like the scrotum, ovaries, bladder, lung, vascular structures, and spermatic cord are rare sites. Scrotal Leiomyoma can develop from smooth muscles of the epididymis, spermatic cord, tunica albuginea, or scrotal wall [1].

It was reported for the first time in 1858 by Forster [2].

Scrotal Leiomyoma is a benign mesenchymal tumor that grows slowly. The Patient with Scrotal Leiomyoma is asymptomatic and presents with only a painless mass, which he has not noticed for years [3,4].

Ultrasound is a first-line and noninvasive method to evaluate patients with scrotal masses in physical examination [5]. For Definitive diagnosing of leiomyoma, histologic examination is advised. In histology, smooth muscle bundles are seen. It is important to differentiate leiomyoma from a malignant type known as leiomyosarcoma [6].

Here in, we report a rare case presentation of a 45-year-old Persian man with solitary scrotal leiomyoma that is confirmed by histology.

## 2 Case presentation

The patient was a 45-year-old Persian man without any medical or surgical history, who presented with a scrotal mass to the urology department of our hospital. The patient suddenly realized a painless scrotal mass about 16 months ago. He reported the size of the mass has not increased compared to the first time he noticed it. He also had no history of infertility, cryptorchidism, or Past trauma. No flank pain, dysuria, hematuria, urinary frequency, incontinence, or urgency was present. He didn't have a family history of genitourinary malignancy.

On presentation, he wasn't ill and toxic in general appearance. The vital signs were in the normal range. On physical examination, he had a single, firm, and mobile mass about 2–3 cm in diameter within the right scrotum. The mass is without tenderness. The left side was normal without any mass. He didn't have palpable inguinal lymph nodes. Rest of the systemic examination did not reveal abnormalities.

**Fig. 1:**
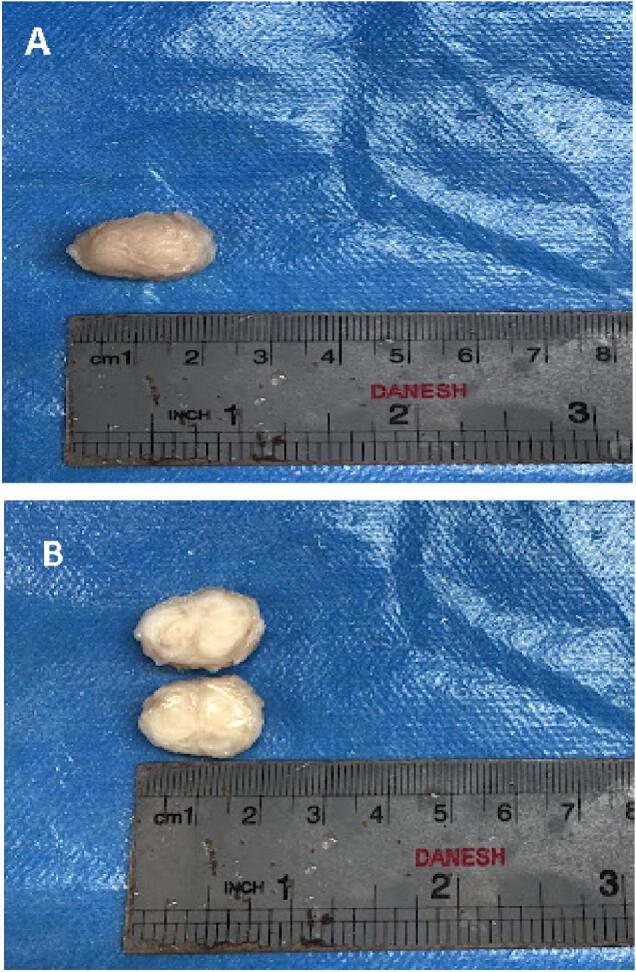
A and B show resected solid mass measuring 2 ∗ 1.5 ∗ 1 cm with creamy–gray color and firm consistency. The cut section shows a homogenous creamy-white color with a whorling appearance.

Laboratory tests were requested for the patient and their findings are shown in Table 1. We asked for pre-operative scrotal sonography that revealed a 2.5 cm hypoechoic extra testicular mass with sharp borders in the right scrotal sac. Then the patient underwent surgery. Intraoperatively a right inguinal incision was done and a well-circumscribed mass with a creamy-white color measuring 2.5 ∗ 1.5 ∗ 1 cm without connection to his testis, epididymis, and spermatic cord was found. The mass was excised totally and the testis was preserved. The resected Specimens were sent to the pathology laboratory. The specimens were cut into multiple pieces in two blocks and 70 % of them were embedded (Fig. 1).

**Table 1:** Laboratory findings of the patient.

Test	Result	Reference range
WBC (10^9^/L)	7.200	4.0–11.0
RBC (10^6^/μL)	5.94	
HB (g/dL)	16.9	13–16
HCT	50.7	
MCV	85.4	
MCH	28.5	
MCHC	33.3	
PLT (10^3^/μL)	254	150–450
BUN (mg/dL)	10	6–20
Cr (mg/Dl)	0.9	0.6–1.3
AST (U/L)	27	<37
ALT (U/L)	32	<41
ALP(U/L)	238	100–360
FBS	73	<100 normal
		100 to 125 prediabetes
		≥126 diabetes
Urinalysis	NEG	
Urine culture	No growth after 48 h	
Anti-HCV Ab	NEG	
HBsAg	NEG	
Blood group and RH	A+

**Fig. 2:**
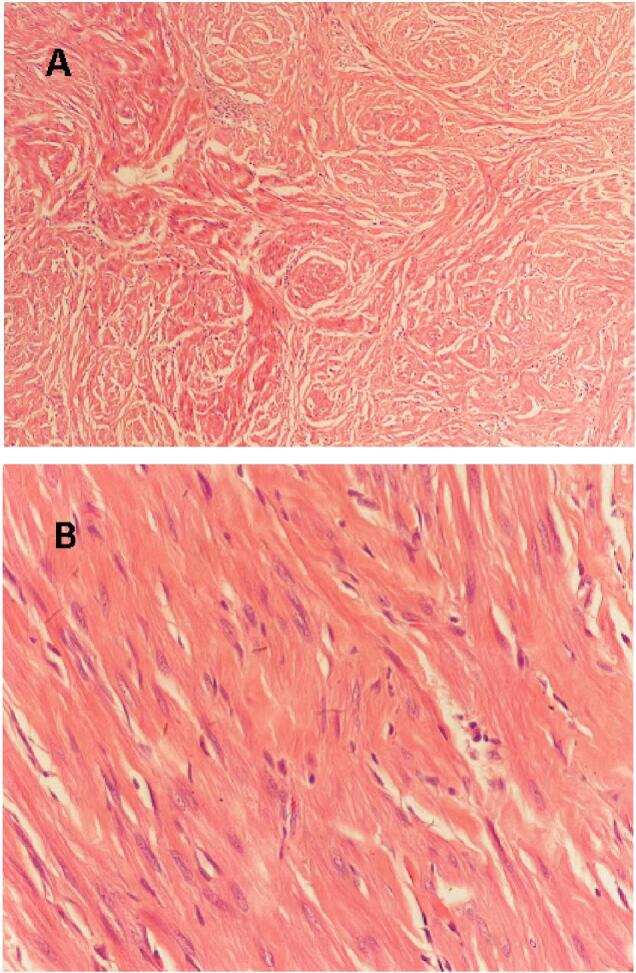
A and B show bland spindle cells with abundant cytoplasm arranged within fibrous and hyalinized connective tissue without nuclear pleomorphism, atypia, mitosis, or invasion by hematoxylin and eosin (H&E) stain.

H&E staining and immunohistochemistry were performed for histopathological examination. On microscopic examination of the scrotal mass revealed interlacing bundles of spindle-shaped cells with no mitotic figures or nuclear pleomorphism (Fig. 2).

**Fig. 3:**
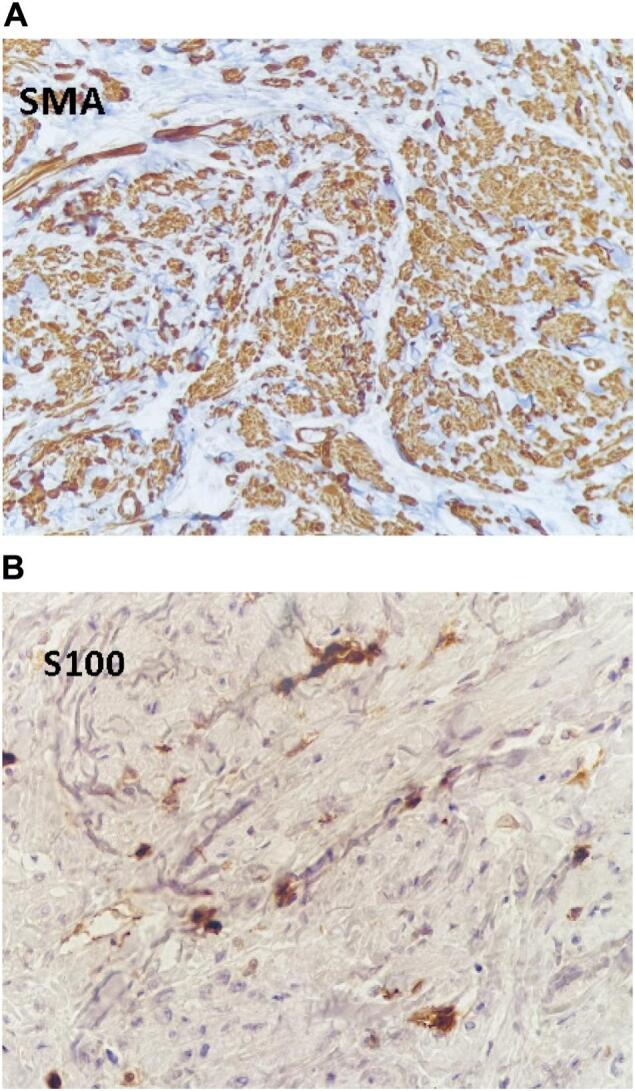
Immunohistochemical analysis show SMA (smooth muscle actin) strong cytoplasmic staining of spindle cells (3A)/S100 staining is negative (3B).

On immunohistochemical analysis, the spindle cells were positive for SMA (smooth muscle actin) and negative for S100, and muscular origin was confirmed (Fig. 3).

Based on the histopathological and immunohistochemical findings, scrotal leiomyoma was diagnosed. This work has been reported in line with the SCARE criteria [7].

## 3 Discussion

In a classification of Scrotal masses, most Solid intratesticular masses are malignant although Extra testicular masses are usually benign and cystic. Lipoma is the most common benign extra testicular solid mass of the scrotum. However, Leiomyoma is a rare extra testicular solid mass that originates from smooth muscles of the epididymis, spermatic cord, tunica albuginea, or scrotal wall [1].

Scrotal Leiomyoma is a benign mesenchymal tumor that grows slowly. The Patient with Scrotal Leiomyoma is asymptomatic and presents with a solitary painless mass, which he has not noticed for years. The size of the tumor is usually <3 cm [3,4]. The patient is asymptomatic with a mass measuring <3 cm and slow growth that he didn't notice.

In some cases, scrotal leiomyoma presents with hydrocele, and in most cases, the tumor biomarkers are normal [8].

Ultrasound is a first-line and noninvasive method to evaluate patients with scrotal masses in physical examination. It can distinguish between intratesticular and extra-testicular scrotal masses. It has a sensitivity of 92 %–98 % sensitivity and 95 %–99.8 % specificity in the diagnosis of testicular malignancy [5,9].

For Definitive diagnosing of leiomyoma, histologic examination is advised. In histology, a well-circumscribed tumor composed of smooth muscle bundles with fibrous and hyalinized connective tissue is seen [10]. It can be confirmed by an Immunohistochemistry (IHC) study. In the IHC study, the smooth muscle cells are positive for vimentin, desmin, smooth muscle actin (SMA), and muscle-specific actin markers staining and negative for S100 staining [6]. SMA is positive in our case spindle cell origin was confirmed and S100 is negative that neurofibroma and schwannoma were rolled out.

The prognosis of scrotal leiomyoma is good [11].

However, it is important to differentiate leiomyoma from malignant course to avoid an over-diagnosis and unnecessary treatments. In histology, the malignant type known as leiomyosarcoma has bizarre nuclei, hypercellularity, and high mitotic activity with infiltrating margins. Also, leiomyosarcoma is larger than leiomyoma. For differentiation between leiomyoma and leiomyosarcoma, an IHC study is advised [6].

After diagnosing this benign mass, surgical management with the goal of organ preservation is advised [5,12]. According to guidelines, small testicular lesion (tumor volume <2.8 cm^3^) with history of hormone disorders (AFP, HCG and LDH) or infertility and long duration of symptoms predict benign histology and testis-sparing surgery (TSS) is recommend [13].

## 4 Conclusion

Scrotal Leiomyoma is a benign and rare mesenchymal tumor in middle-aged men that presents with a slow-growing and painless mass. Scrotal ultrasound is a first-line and noninvasive method to evaluate patients that is confirmed by histologic examination. It is important to differentiate leiomyoma from malignant course.

## List of abbreviations

H&E: hematoxylin and eosin
IHC: immunohistochemistry
SMA: smooth muscle actin

## Author contribution

M. Karimi and H. Raad participated in the conception and design of the report.

M. Karimi wrote the manuscript and evaluated the patient. M. Karimi and H. Rad wrote the pathology report.

Two authors reviewed the manuscript and approved the final manuscript.

## Consent for publication

Written informed consent was obtained from the patient for publication of this case report and any accompanying images. A copy of the written consent is available for review by the Editor-in-Chief of this journal.

## Ethical approval

Hormozgan University of Medical Sciences Ethical Committee approved the study under the ethical code IR.HUMS.REC. 1401.236 and the study conforms with the Helsinki Declaration's statements.

## Guarantor

Mohadeseh Karimi.

## Research registration number

Heterotopic abdominal wall ossification: a case report.

researchregistry9814.

## Funding

The study did not receive any funding.

## Conflict of interest statement

The authors declare no conflict of interest.

## Data Availability

The data sets used during the current study are available from the corresponding author upon reasonable request.
